# Salinity Effect in Seawater Thermoelastohydrodynamic Lubrication of Double Spiral Groove Face Seals

**DOI:** 10.3390/ma19020285

**Published:** 2026-01-09

**Authors:** Shaoxian Bai, Demin Yang, Jing Yang

**Affiliations:** College of Mechanical Engineering, Zhejiang University of Technology, Hangzhou 310032, China; yangdemin2025@163.com (D.Y.); yangjing@zjut.edu.cn (J.Y.)

**Keywords:** salinity effect, seawater, thermoelastohydrodynamic lubrication, double spiral grooves, face seal

## Abstract

A rise in seawater salinity results in an increase in its viscosity, which presents a coupled influence on the distribution of fluid pressure, temperature and deformation at the sealing face, leading to fluctuations in sealing performance and forming the salinity effect in seawater thermoelastohydrodynamic lubrication (TEHL). Here, for a double spiral groove face seal, a TEHL model is established and numerical analysis is carried out, taking account of the salinity effect and cavitation effect, with the aim to ensure that the seal maintains stable performance under varying conditions of sea depth and speed. It is found that the effect of salinity on the opening force and leakage rate exhibits obvious nonlinear variations. As salinity rises from 0 to the standard 35 g/kg, the opening force changes by about 5%, and there is a transition between forward and reverse leakage, with variations of approximately ±100%. More importantly, the double spiral grooves offer the potential for a zero-leakage design in seawater face seals, even under pressures exceeding 4 MPa, through precise design. Additionally, the double spiral groove face seal shows excellent adaptability under multipoint conditions and can facilitate a zero-leakage design in varying pressure, speed and temperature conditions. This provides theoretical support for deep-sea equipment and applications in other extreme environments.

## 1. Introduction

Due to the ocean’s rich resources, it has become a very important energy base and a strategic space for sustainable development for mankind. However, the deep sea is a very harsh environment due to the ultra-high pressure, low temperature, darkness and salinity. These complex working conditions lead to many challenges in the structural design of marine equipment. As a core component in maintaining the stability and reliability of marine equipment such as ship drive shafts and diesel engines, mechanical seals need to withstand deep-sea pressure and temperature vibration. With the temperature ranging from −2 °C to 30 °C and the pressure ranging from zero to several MPa in field applications of seawater seals, a new problem of seawater salinity arises in low-leakage and high-reliability designs, since the density and viscosity of seawater are sensitive not only to temperature and pressure but also to salinity, leading to more complex TEHL behaviors. However, relatively few research works have focused on the influence of salinity on TEHL behavior.

In recent decades, non-contact sealing technology has developed rapidly with the proposal of various types of upstream pumping grooves [1,2,3,4,5,6,7,8], such as spiral grooves (Lai, 1994 [1]; Warda et al., 2018 [3]; Xiao and Khonsari, 2012 [4]; Medjahed et al., 2023 [5]; Song et al., 2023 [6]), linear grooves (Lebeck, 2008 [2]) and ellipse grooves (Bai et al., 2022 [7]), offering greater possibility for the realization of zero-leakage and long-service-life seal designs. However, they largely rely on the precise force balance design of the sealing face through TEHL analysis, because the high seal pressure and temperature rise cause significant face deformation, often leading to the disruption of this force balance (Bai and Wen, 2019 [8]). For instance, for a spiral groove face seal, the convergent face deformation may reach 3 μm with a speed of 2000 rpm and pressure of 2 MPa, and the leakage rate increases by 50 times (Xiao and Khonsari, 2012 [4]; Song et al., 2023 [6]). A series of TEHL works on the taper-type (Ran et al., 2024 [9]; Srivastava et al., 2019 [10]), triangular dimple textures (Adjemout, 2018 [11]), wavy surface (Su, 2023 [12]) and semi-circular grooves (Liang et al., 2024 [13]) also proved that the face deformation often makes the film thickness and leakage rate increase.

Now, with the expanded application of mechanical seals across more fields, including closed-loop supercritical CO_2_ [14,15], precooling technology for supersonic aeroengines [16,17] and reactor coolant pumps [12,18], the properties of sealing fluids have become increasingly important in the TEHL design of face seals. For instance, supercritical CO_2_ presents a drastic change in viscosity (Chupp et al., 2006 [19]; Wang et al., 2020 [20]), which leads to a significant variation in the sealing performance, including a decrease in the opening force by 10% and a sharp increase in the leakage rate by 10 times in the TEHL analysis (Fairuz and Jakn, 2016 [21]; Fairuz et al., 2019 [22]; Zhu and Bai, 2021 [23]). When the properties of the fluid and temperature are coupled, it often leads to more drastic changes in sealing performance. A mutational phenomenon of both temperature and pressure occurs near 250 K in the TEHL analysis of a Helium spiral-grooved face seal. There, the leakage varies by more than 50% due to the increase in the temperature gradient (Wang and Bai, 2022 [24]). More importantly, the transformation between the convergent deformation and the divergent occurs on the sealing face (Ma and Bai, 2025 [25]), which requires a more accurate TEHL analysis during the sealing design.

Due to the incompressibility of liquids and the fact that the viscosity of liquids is much greater than that of gases, not only does the seal have a significant dynamic pressure effect, but its leakage rate also shows a complex variation because of the face deformation (Ran et al., 2024 [9]; Yang et al., 2025 [26]). For a liquid spiral groove face seal, the face deformation may result in an increase of 68% for the leakage rate at a speed of 10,000 rpm and a pressure of 0.3 MPa (Bai et al., 2022 [7]). For seawater, the salinity causes significant changes in fluid properties (Isdale and Morris, 1972 [27]; Sharqawy et al., 2010 [28]; Jamieson et al., 1969 [29]). When the salinity increases from 0 to 35 g/kg at a temperature of 300 K, the viscosity increases by about 0.5%. In contrast, for standard seawater with a salinity of 35 g/kg, the viscosity presents a decrease of about 80% when the temperature increases from 300 K to 450 K (Jamieson et al., 1969 [29]). Theoretically, the salinity will have an obvious impact on the TEHL performance of seawater seals, especially on the leakage.

This study intends to investigate the salinity effect on TEHL behaviors of seawater face seals, which can ensure that the designed seal structure maintains a stable sealing performance under varying conditions of sea depth and speed. All equations used in this numerical model are solved by a finite difference method and finite element method after programming. For a double spiral groove face seal, the salinity effect on sealing performance is analyzed. Coupled with the properties of seawater, the sealing performance is numerically calculated under varying pressure, speed and temperature conditions. It provides a potential method for the engineering application of zero-leakage seawater face seals.

## 2. Model

**Figure 1:**
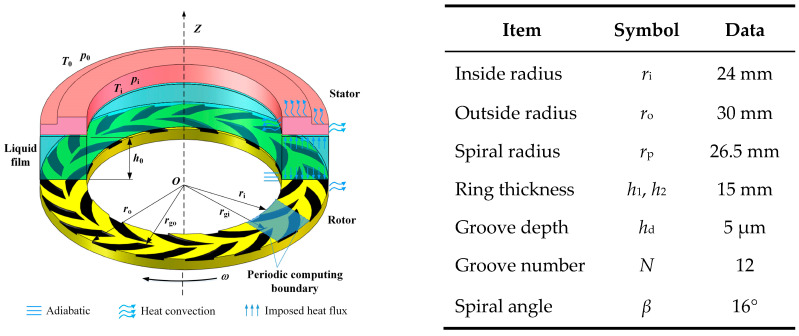
Schematic diagram of a liquid face seal with double spiral grooves.

Figure 1 shows a double spiral groove face seal in a polar coordinate system composed of radial coordinate *r* and circumferential coordinate *θ*. There are two rows of spiral grooves on the rotor face: outer ones and inner ones. At a rotational speed *ω*, the outer grooves produce enough opening force to keep a clearance *h*_0_; meanwhile, the fluid is pumped from the outer radius side to the inner radius side, forming a positive leakage rate. On the other hand, the inner grooves generate an upstream pumping effect, where the fluid is pumped from the inner radius to the outer radius, simultaneously enhancing the opening force.

Generally, as shown in Figure 1, the fluid on the outer diameter side is called the sealed fluid. Due to the pressure of sealed fluid being higher than the ambient pressure at the inner diameter, the sealed fluid flows to the inner diameter side, forming a sealed leakage, which is known as forward leakage. When the upstream pumping effect of the spiral grooves is strong enough, the values of the calculated leakage rate are negative, which means that the fluid flows from the ambient side at the inner diameter to the outer diameter, forming a reverse leakage. There will be no leakage of the sealed fluid, that is, zero leakage.

Since the grooves are uniformly distributed along the circumferential direction, to reduce the computational load, a periodic groove group is taken as the research object to treat the face seal, as shown in Figure 1.

### 2.1. Seawater Properties

According to the TEHL theory, the parameters that affect lubrication performance mainly include density, viscosity, heat capacity and other parameters. For seawater, salinity has become a new influencing factor. Here, the density of seawater, *ρ* with unit kg/m^3^, is calculated using the following equation (Isdale and Morris, 1972 [27]):

(1) $$\rho =10^{3}\left(A_{1}F_{1}+A_{2}F_{2}+A_{3}F_{3}+A_{4}F_{4}\right)$$

where

(2) $$A_{1}=4.032G_{1}+0.115G_{2}+3.26\times 10^{-4}G_{3}$$

(3) $$A_{2}=-0.108G_{1}+1.571\times 10^{-3}G_{2}-4.23\times 10^{-4}G_{3}$$

(4) $$A_{3}=-0.012G_{1}+1.74\times 10^{-3}G_{2}-9\times 10^{-6}G_{3}$$

(5) $$A_{4}=6.92\times 10^{-4}G_{1}-8.7\times 10^{-5}G_{2}-5.3\times 10^{-5}G_{3}$$

(6) $$G_{1}=0.5,G_{2}=B,G_{3}=2B^{2}-1,B=\left(2S_{p}-150\right)/150$$

(7) $$F_{1}=0.5,F_{2}=A,F_{3}=2A^{2}-1,F_{4}=4A^{4}-3A,A=\left(2T-473.15\right)/160$$

*T* is temperature with unit K, and *Sp* is salinity with unit g/kg.

**Figure 2:**
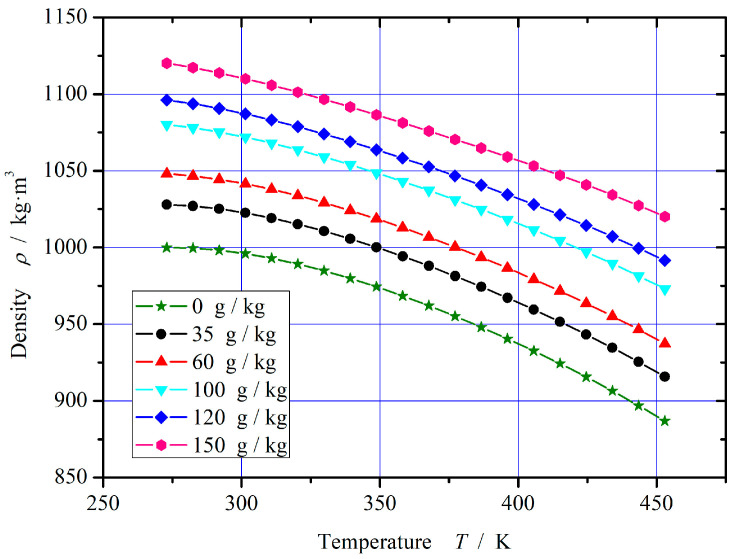
Variation in density with increase in temperature and salinity.

Figure 2 presents the influence of temperature and salinity on seawater density. Clearly, with increasing temperature, the density decreases significantly. Meanwhile, with increasing salinity, the density increases obviously. When the salinity increases from 0 to 35 g/kg at a temperature of 300 K, the density increases by about 2.5% from 997 kg/m^3^ to 1022 kg/m^3^. In contrast, for standard seawater with a salinity of 35 g/kg, when the temperature increases from 300 K to 450 K, the density decreases by about 10% from 1022 kg/m^3^ to 918 kg/m^3^.

The viscosity of seawater, *η* with unit Pa.m/s, is calculated using the following equation (Sharqawy et al., 2010 [28]):

(8) $$\eta =\eta_{w}\left(1+AS_{p}+BS_{p}^{2}\right)$$

where

(9) $$A=1.541\times 10^{-3}+1.998\times 10^{-5}T-9.52\times 10^{-8}T^{2}$$

(10) $$B=7.974\times 10^{-6}-7.561\times 10^{-8}T+4.724\times 10^{-10}T^{2}$$

(11) $$\eta_{w}=4.2844\times 10^{-5}+{\left(0.157{\left(T+64.993\right)}^{2}-91.296\right)}^{-1}$$

**Figure 3:**
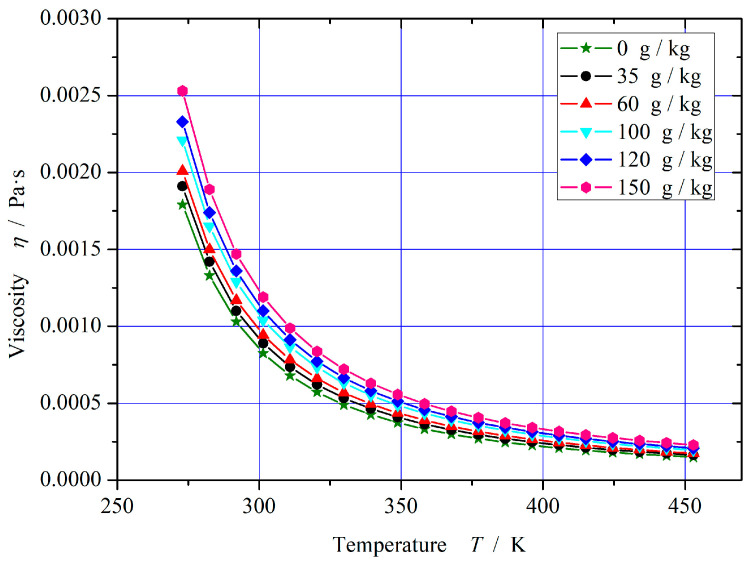
Variation in viscosity with temperature and salinity.

Figure 3 shows the change in seawater viscosity with increasing temperature and salinity. Obviously, with increasing temperature, the viscosity decreases sharply. Meanwhile, with increasing salinity, the viscosity increases slightly. When the salinity increases from 0 to 35 g/kg at a temperature of 300 K, the viscosity increases by about 1.0% from 8.0 × 10^−4^ Pa·s to 8.1 × 10^−4^ Pa·s. In contrast, for standard seawater with a salinity of 35 g/kg, when the temperature increases from 300 K to 450 K, the viscosity decreases by about 80% from 8.1 × 10^−4^ Pa·s to 1.5 × 10^−4^ Pa·s.

The specific heat capacity of seawater, *C*_v_ with unit kJ/(kg.K), is calculated using the following equation (Jamieson et al., 1969 [29]):

(12) $$C_{v}=A+BT+CT^{2}+DT^{3}$$

where

(13) $$A=5.328-9.76\times 10^{-2}S_{p}+4.04\times 10^{-4}{S_{p}}^{2}$$

(14) $$B=-6.913\times 10^{-3}+7.351\times 10^{-4}S_{p}-3.15\times 10^{-6}{S_{p}}^{2}$$

(15) $$C=9.6\times 10^{-6}-1.927\times 10^{-6}S_{p}+8.23\times 10^{-9}{S_{p}}^{2}$$

(16) $$D=2.5\times 10^{-9}+1.666\times 10^{-9}S_{p}+7.125\times 10^{-12}{S_{p}}^{2}$$

**Figure 4:**
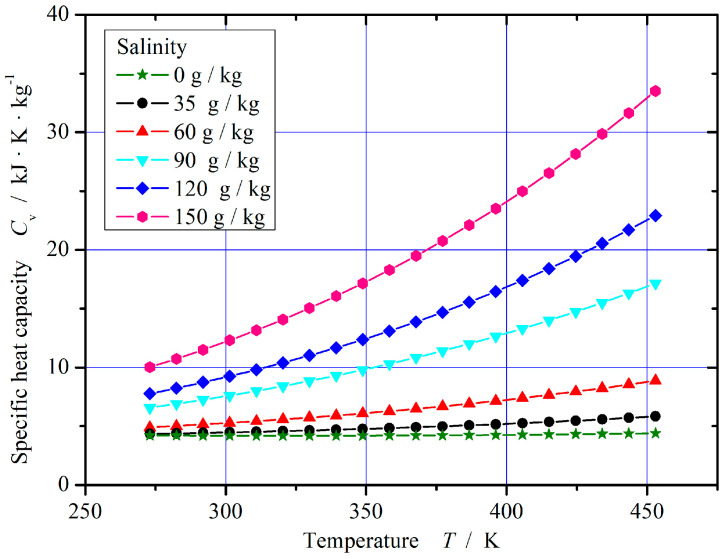
Change in specific heat capacity with temperature and salinity.

Figure 4 presents the curves of the specific heat capacity with increasing temperature and salinity. It can be seen that with increasing temperature as well as salinity, the specific heat capacity increases. For standard seawater with a salinity of 35 g/kg, when the temperature increases from 300 K to 450 K, the specific heat capacity increases by about 10% from 4.5 kJ·K/kg to 5.8 kJ·K/kg.

### 2.2. Control Equations

The steady-state Reynolds equation for seawater lubrication is as follows:

(17) $$\frac{𝜕}{r𝜕\theta }\left(\frac{\rho h^{3}}{\eta }\frac{𝜕p}{r𝜕\theta }\right)+\frac{𝜕}{r𝜕r}\left(\frac{\rho h^{3}r}{\eta }\frac{𝜕p}{𝜕r}\right)=6\omega \frac{𝜕\left(\rho h\right)}{𝜕\theta }$$

The fluid temperature *T* on sealing faces can be obtained by solved the following energy equation:

(18) $$\begin{array}{l} \left(\frac{h^{3}}{12\eta }\frac{𝜕p}{r𝜕\theta }-\frac{\omega rh}{2}\right)\frac{𝜕T}{r𝜕\theta }+\left(\frac{h^{3}}{12\eta }\frac{𝜕p}{𝜕r}\right)\frac{𝜕T}{𝜕r}\\ =-\frac{\eta \omega^{2}r^{2}}{h\rho c_{v}}+\frac{h^{3}}{12\eta \rho c_{v}}\left[{\left(\frac{𝜕p}{r𝜕\theta }\right)}^{2}+{\left(\frac{𝜕p}{𝜕\theta }\right)}^{2}\right]-\frac{k_{gs1}}{\rho c_{v}}\left(T_{s1}-T\right)-\frac{k_{gs2}}{\rho c_{v}}\left(T_{s2}-T\right) \end{array}$$

where *k*_gs1_ and *k*_gs2_ represent the convective heat transfer coefficient between the rings and fluid film, respectively; *T*_s1_ and *T*_s2_ denote the surface temperatures of the stator and rotor, respectively.

The ring temperature *T*_s_ can be obtained by solving the thermal conductive equation. For the rotor ring, its expression is as follows:

(19) $$\frac{{𝜕}^{2}T_{s}}{r^{2}𝜕\theta^{2}}+\frac{𝜕}{r𝜕r}\left(r\frac{𝜕T_{s}}{𝜕r}\right)+\frac{{𝜕}^{2}T_{s}}{𝜕z^{2}}=0$$

For the stator ring with a thermal conductive coefficient *k*_c2_, density *ρ*_s2_ and specific heat capacity *c*_s2_, its expression is as follows:

(20) $$\frac{k_{c2}}{\rho_{s2}c_{s2}}[\frac{{𝜕}^{2}T_{s}}{r^{2}𝜕\theta^{2}}+\frac{1}{r}\frac{𝜕}{𝜕r}(r\frac{𝜕T_{s}}{𝜕r})+\frac{{𝜕}^{2}T_{s}}{𝜕z^{2}}]=\omega \frac{𝜕T_{s}}{𝜕\theta }$$

The heat flux that occurs between the liquid film and sealing rings can be depicted by the interface equations below:

(21) $$-k_{c1}{\left(\frac{𝜕T_{S}}{𝜕n}\right)}_{s}=k_{\mathrm{gs}1}\left(T_{s1}-T\right)$$

(22) $$-k_{c1}{\left(\frac{𝜕T_{S}}{𝜕n}\right)}_{s}=k_{\mathrm{gs}2}\left(T_{s2}-T\right)$$

where *k*_c1_ is the thermal conductivity of the rotor ring.

### 2.3. Cavitation

The cavitation effect is also considered in the TEHL analysis by using the following density equation (Yang et al., 2025 [26]).

(23) $$\left\{\begin{array}{cc} \rho =\rho_{0} & \begin{array}{cc} \mathrm{if} & p>p_{c} \end{array}\\ \frac{\rho }{Tp}=\frac{\rho_{0}}{T_{c}p_{c}} & \begin{array}{cc} \mathrm{if} & p\leq p_{c} \end{array} \end{array}\right.$$

where *p*_c_ is the critical cavitation pressure when the cavitation occurs with the density *ρ*_0_ of the liquid state at the local temperature *T*_c_. Here, for the water and the seawater, the value of critical cavitation pressure is set to 0.1 MPa.

### 2.4. Boundary Conditions

In order to solve the numerical model, the following pressure boundary conditions are used:

(24) $$p(r=r_{i},\theta )=p_{i}$$

(25) $$p(r=r_{o},\theta )=p_{o}$$

(26) p(r,θ=π/N)=p(r,θ=−π/N)

where Equations (24) and (25) are the compulsory pressure boundary conditions for the double spiral groove face seal. Since the spiral grooves are periodically distributed along the circumferential direction, a period domain is selected as the numerical calculation region to reduce the computational difficulty. The periodic pressure boundary condition is defined as Equation (26).

### 2.5. Numerical Method

To acquire the film pressure, film temperature and ring temperature, the finite difference method is employed. For the coupling calculation of the face deformations, the finite element method is applied.

**Figure 5:**
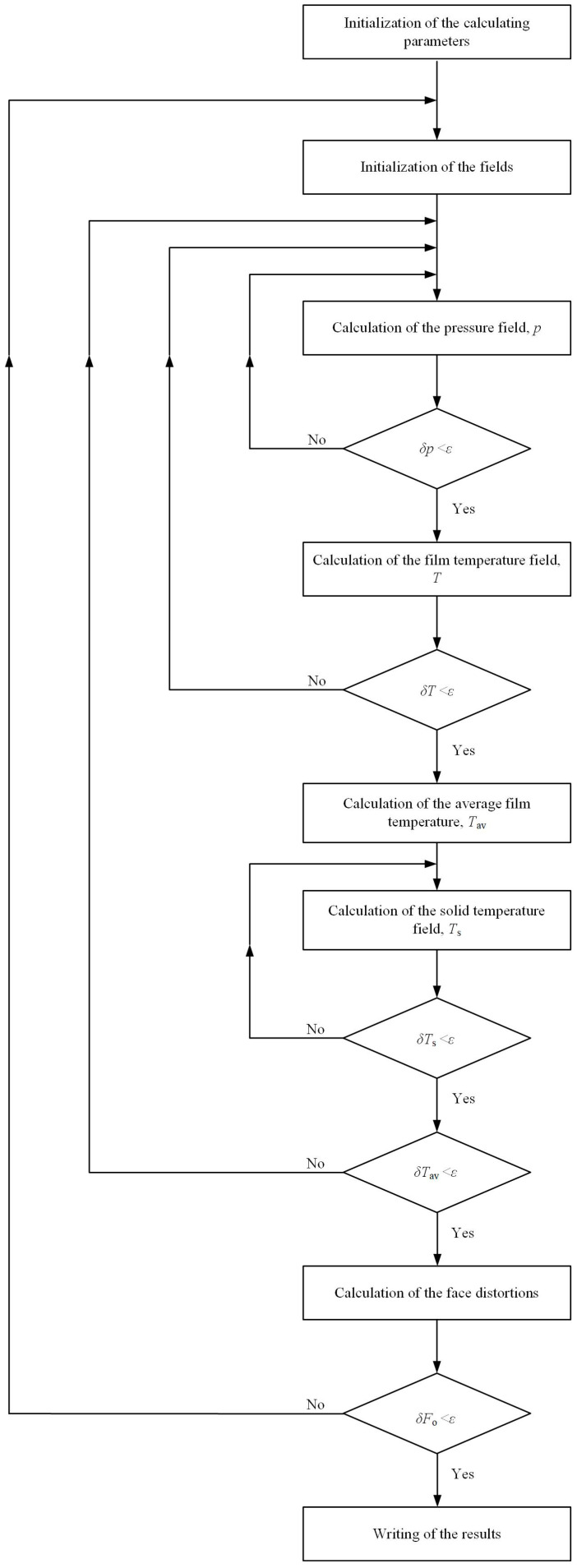
Flowchart of the numerical procedure.

The mesh density for the liquid film is 60 × 61, and for the rings, it is 60 × 61 × 25. In the convergence criterion, the error limit, *e*, is set to 10^−5^. As depicted in Figure 5, after all the parameters are initialized, the film pressure and film temperature are obtained based on the governing equation. When the temperature distribution is converged, the average film temperature distribution will be obtained. Based on the average distribution, we will calculate the ring temperature, opening force and face deformation. The whole calculation process will end when the face deformation is converged.

In order to characterize the sealing performance, the dimensionless opening force is defined as

(27) $$F_{\mathrm{open}}=\frac{1}{p_{a}r_{i}^{2}}\int_{0}^{2\pi }\int_{r_{i}}^{r_{o}}prd\theta dr$$

The dimensionless leakage rate is defined as

(28) $$Q=\frac{1}{h_{0}^{3}p_{a}}\int_{0}^{2\pi }h^{3}r\frac{𝜕p}{𝜕r}d\theta$$

The material parameters of the seawater face seal are listed in Table 1.

## 3. Model Validation

**Figure 6:**
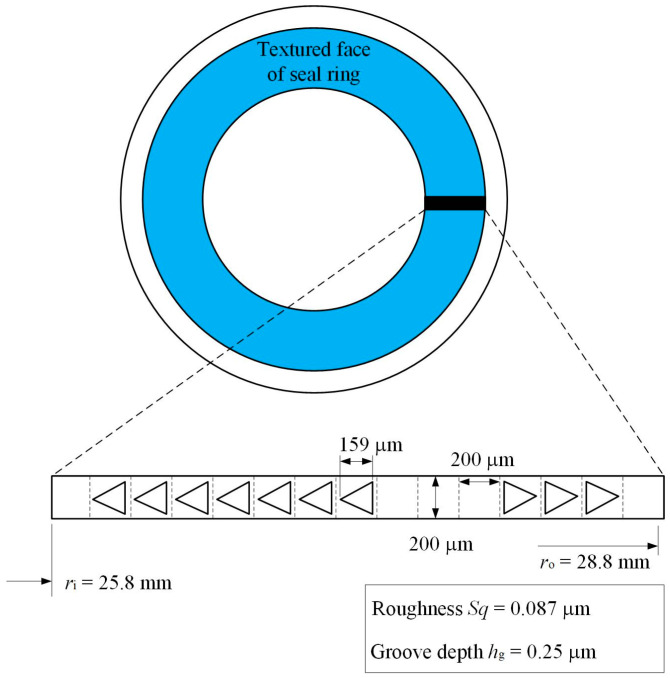
Illustration of triangular dimple surface in Dinggui’s experimental work [30].

The experimental work conducted by Dinggui [30] on triangular dimple surface texturing, as shown in Figure 6, is further utilized to validate the proposed model.

**Figure 7:**
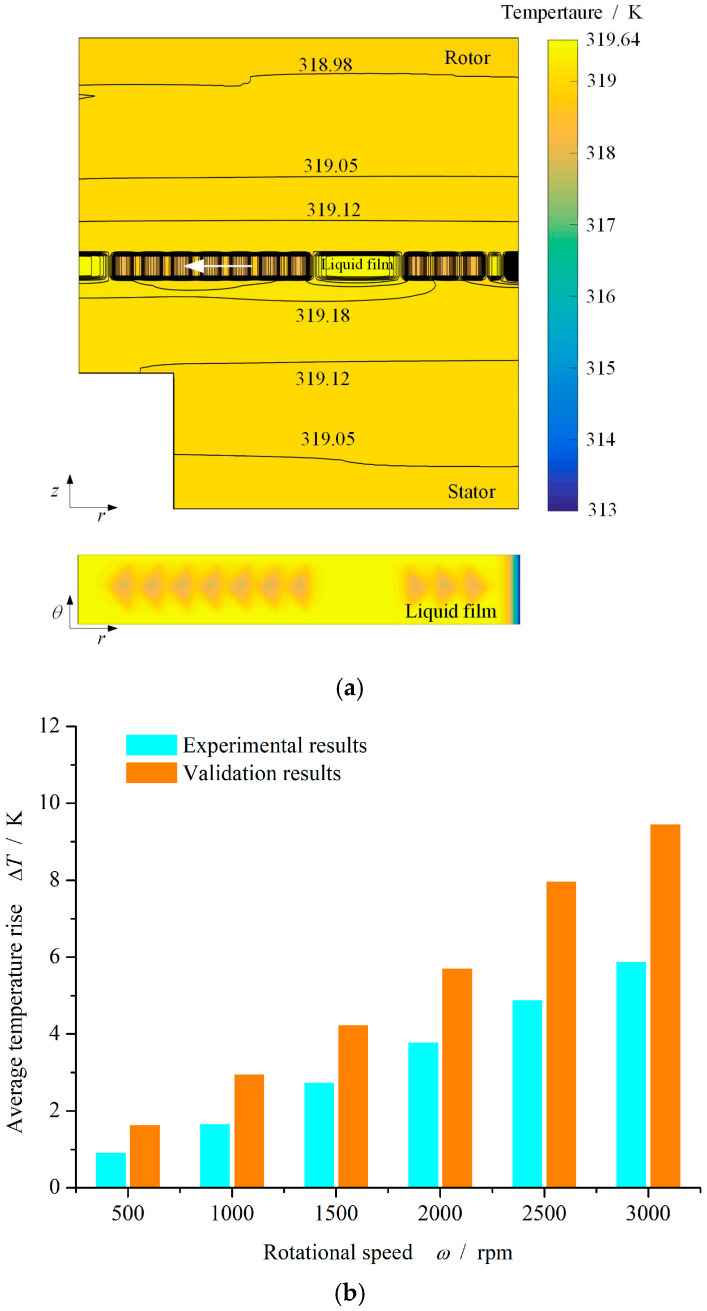
Comparison temperature between experimental work and proposed model (*p*_0_ = 0.6 MPa, *p*_i_ = 0.1 MPa, *T* = 313 K). (**a**) Temperature distribution (*w* = 2000 rpm) and (**b**) average temperature rise.

The comparison results shown in Figure 7 indicate that the present theoretical model can predict temperature changes quite well and can be further applied to the following TEHL analysis of seawater seals.

## 4. Salinity Effect

**Figure 8:**
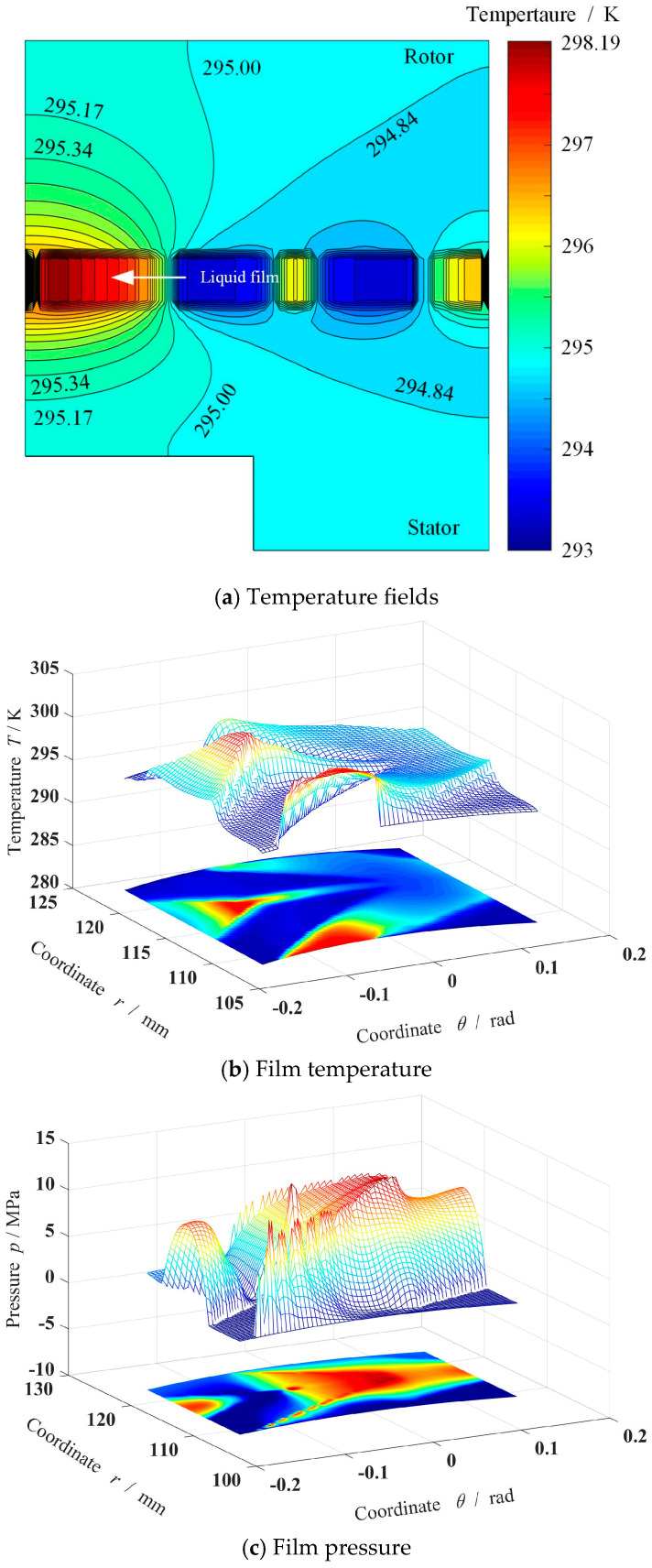
The temperature and pressure distributions of double spiral groove face (*p*_o_ = 2.6 MPa, *p*_i_ = 0.1 MPa, *T* = 293 K, *w* = 10,000 rpm, *S_p_* = 35 g/kg).

Figure 8a shows the temperature distribution of the sealing film and rings. It is demonstrated that both the rotor and the stator exhibit a notable temperature rise of approximately 5 K. Significantly, there exists an evident temperature gradient within the rings, with an approximate increase of 2.3 K for both the rotor and the stator from the outer radius to the inner, indicating the presence of thermal convergent deformation on the sealing faces.

Further, the upstream pumping effect of the inner groove results in higher fluid pressure. Obviously, the maximum pressure occurs at the center part of the ring face, where the pumped fluid caused by the outer spiral groove and that of the inner spiral groove are simultaneously squeezed, forming a pressure value of 12.2 MPa, much higher than the seal pressure of 2.6 MPa.

**Figure 9:**
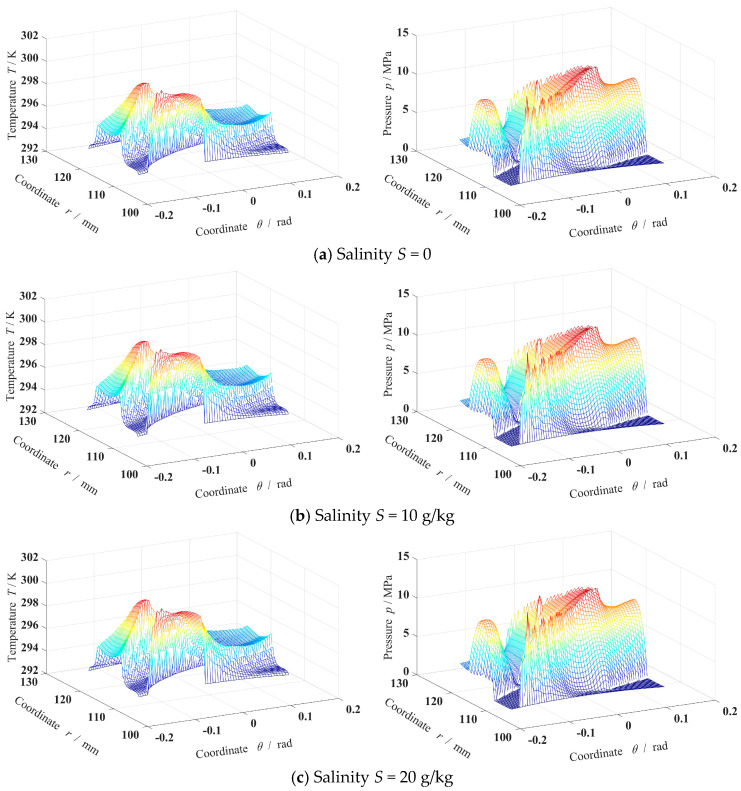

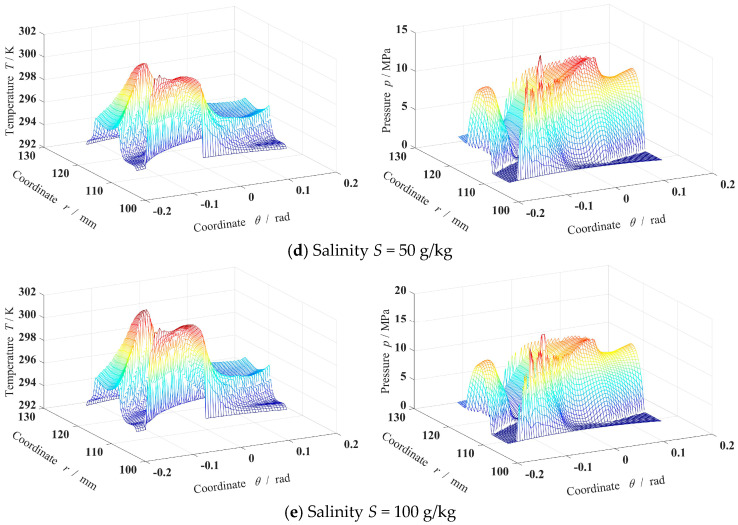
Influence of salinity on temperature and pressure distributions (*p*_o_ = 2.6 MPa, *T* = 293 K, *w* = 10,000 rpm, *S_p_* = 35 g/kg).

Theoretically, for the liquid face seals, when the liquid leaks from regions of higher pressure to those of lower pressure, it is often accompanied by an increase in temperature. Here, as illustrated in Figure 8c, for this face with double spiral grooves, there are two high-temperature parts in the fluid film. One is situated adjacent to the inner diameter, while the other is located at the middle face. However, overall, the temperature near the inside diameter is higher than that near the outside. Therefore, a convergent deformation will be produced, which aligns well with published works. For seawater, salinity is a parameter that cannot be ignored due to its obvious effects on fluid properties, as discussed in the section above. Figure 9 presents the influence of salinity on temperature and pressure distributions. As illustrated in the figure, the maximum film temperature experiences a slight increase from 297 K to 300 K as salinity rises from 0 to 100 g/kg. Concurrently, the maximum film pressure rises from 11.3 MPa to 15.3 MPa.

**Figure 10:**
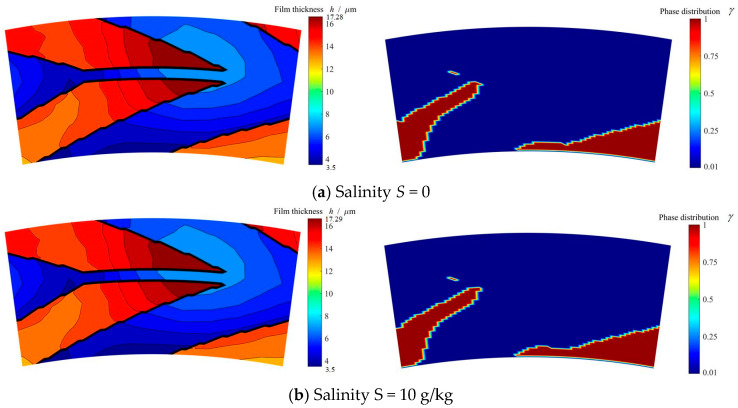

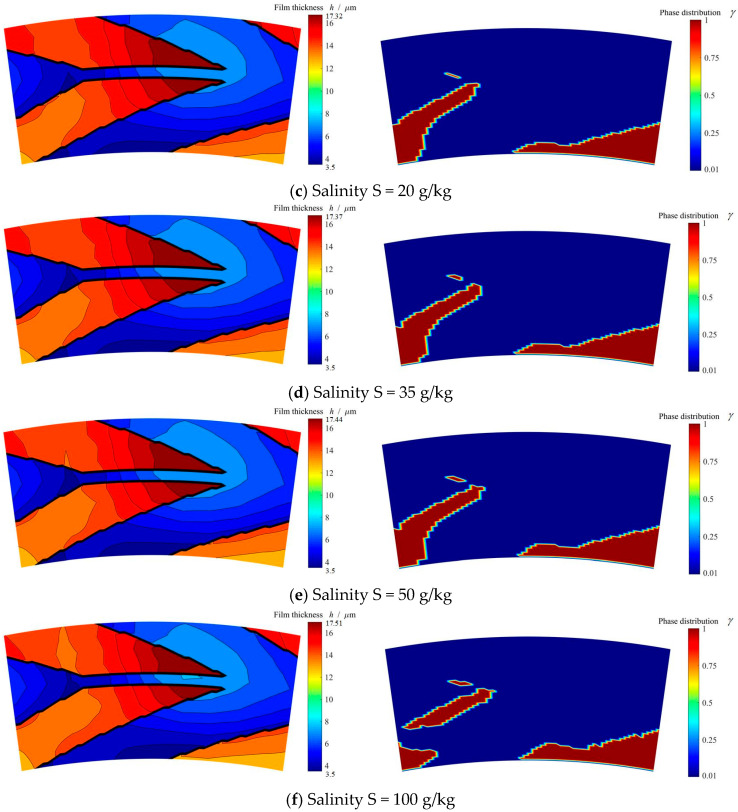
Influence of salinity on film thickness and cavitation distributions (*p*_o_ = 2.6 MPa, *p*_i_ = 0.1 MPa, *T* = 293 K, *w* = 10,000 rpm, *S_p_* = 35 g/kg).

The temperature gradient often leads to thermal deformation coupled with mechanical deformation. As shown in Figure 10, the face deformation presents a convergent clearance. On the other hand, the maximum deformation occurs near the ends of spiral grooves because of the high-pressure peaks. The maximum value of face deformation increases with increasing salinity, 4.11 μm at salinity *S_p_* = 0, 4.33 μm at salinity *S_p_* = 35 g/kg and 4.59 μm at salinity *S_p_* = 100 g/kg.

Another important phenomenon is the cavitation, which affects sealing performance significantly. It can be seen in Figure 10 that the cavitation mainly occurs in the inner grooves near the low-pressure side. With increase in salinity, the profile of the cavitation area changes slightly.

**Figure 11:**
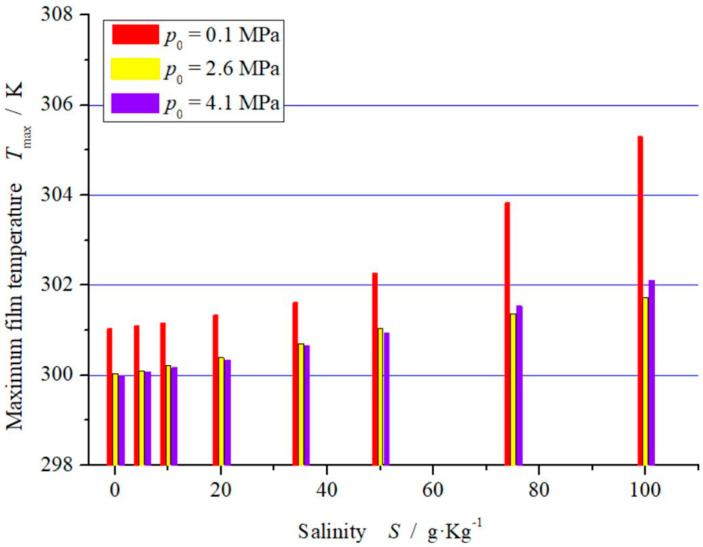
Influence of salinity on maximum film temperature (*T*_o_ = 293 K, *w* = 10,000 rpm).

Briefly, the influence of salinity on seawater sealing is directly manifested as temperature rise and changes in sealing performance. Figure 11 shows that the maximum film temperature presents a continuous increasing trend with increasing salinity. Obviously, the temperature rise under high-seal-pressure conditions (*p*_o_ = 2.6 MPa and 4.1 MPa) is significantly lower than that under zero seal pressure condition (*p*_o_ = 0.1 MPa). The reason is that under high-pressure conditions, the increase in leakage rate takes away more heat. Moreover, due to the large specific heat capacity and low viscosity of seawater, the overall temperature rise of the seal is relatively small, within a few degrees.

**Figure 12:**
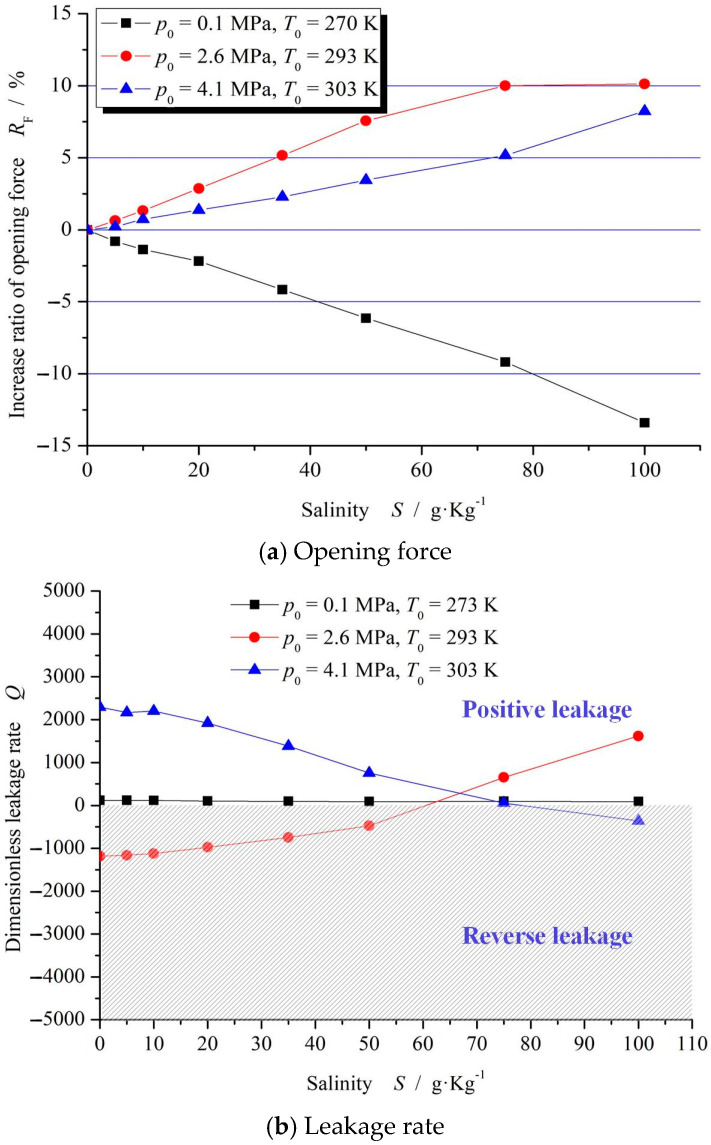
Salinity effect on sealing performance (*w* = 10,000 rpm).

Although the influence of salinity on deformation is at the sub-micron level, due to the fact that the sealing performance is highly sensitive to changes in the gap, the variation in sealing performance with salinity is very significant. As shown in Figure 12, the opening force presents complex trends with the increase in salinity. In the case of zero seal pressure of *p*_o_ = 0.1 MPa, the dimensionless opening force decreases by approximately 4% from 8.93 to 8.56 when the salinity rises from 0 to 35 g/kg. Conversely, for the case of high seal pressure of *p*_o_ = 2.6 MPa, the dimensionless opening force increases by about 5% from 40.64 to 42.74 as the salinity increases from 0 to 35 g/kg.

Correspondingly, the leakage rate presents different trends for high seal pressure cases of *p*_o_ = 2.6 MPa and *p*_o_ = 4.1 MPa. When the seal pressure is *p*_o_ = 2.6 MPa, the value of the leakage rate shifts from negative to positive with increase in salinity at *S_p_* = 60 g/kg. In contrast, for the case of the seal pressure of *p*_o_ = 4.1 MPa, the leakage rate presents an opposite change with increasing salinity at *S_p_* = 75 g/kg. Here, the negative values of leakage rate mean that the seal reaches a full upstream pumping state of zero leakage. Generally, this is induced by the inner spiral grooves. In contrast, the positive values of leakage rate mean that the downstream pumping effect induced by the outer spiral grooves is stronger than the upstream pumping effect induced by the inner ones.

**Figure 13:**
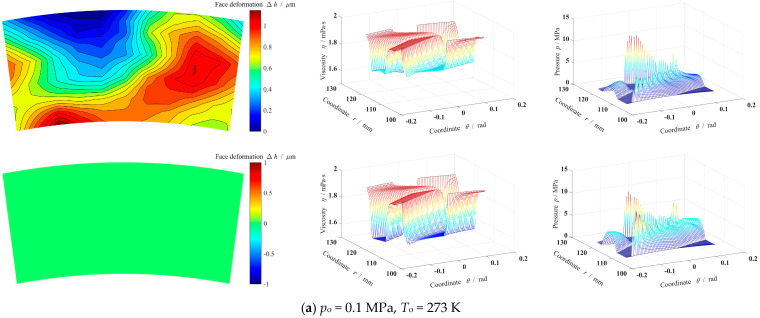

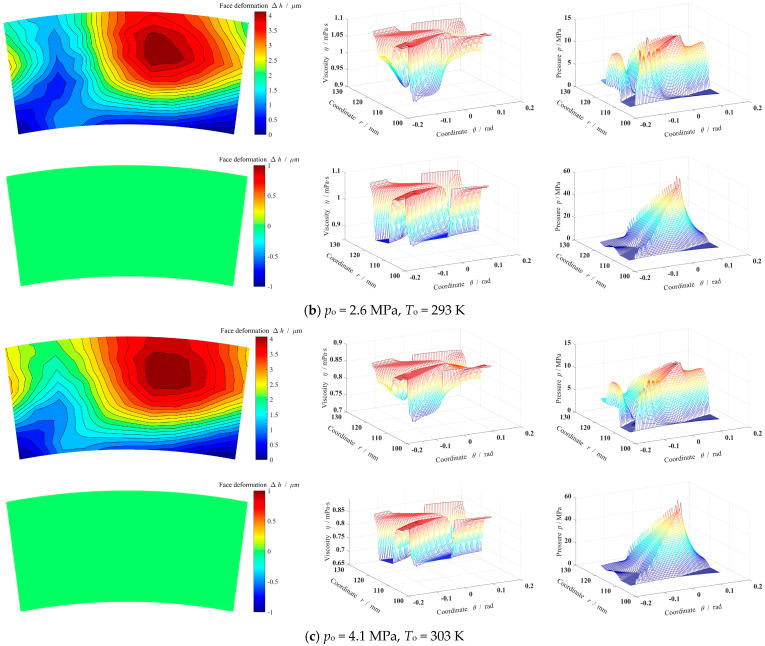
Influence of face deformation on film pressure distribution (*S* = 35 g/kg, *w* = 10,000 rpm).

Another important conclusion is that, as seen in Figure 12, as the temperature and pressure increase, the sealing opening force undergoes a transformation from an increasing trend to a decreasing trend. The main reason is the transformation of face deformation. Figure 13 shows the influence of end face deformation on the pressure distribution. When the seal pressure increases from 0.1 MPa to 2.6 MPa and the seal temperature increases from 273 K to 293 K, the face deformation transfers from divergence to convergence. In the case of seal pressure of 0.1 MPa and temperature of 273 K, there is a maximum deformation value of about 1.4 μm at the inner diameter of the seal ring. In contrast, there is a maximum deformation value of about 4 μm near the outer diameter of the seal ring. Further, the face deformation presents an obvious influence on film pressure distribution. For the case of 0.1 Mpa, the face deformation causes the maximum film pressure to increase from 1.02 MPa to 1.11 MPa. However, for the case of 2.6 MPa, the face deformation results in a significant decrease in maximum film pressure from 5.70 MPa to 1.22 MPa.

Overall, according to the above TEHL analysis, salinity has a significant effect on sealing performance, especially in controlling the leakage rate. Meanwhile, the double spiral grooves enable a zero-leakage design for seawater face seals.

## 5. Sealing Performance

Nowadays, seals are desired to keep zero leakage working in multi-cases of temperature, speed and pressure. In this section, the sealing performance of standard seawater with *S_p_* = 35 g/kg is numerically calculated compared with pure water with *S_p_* = 0 for the double spiral groove face seal under varying ambient temperature, seal pressure and speed conditions.

### 5.1. Ambient Temperature

**Figure 14:**
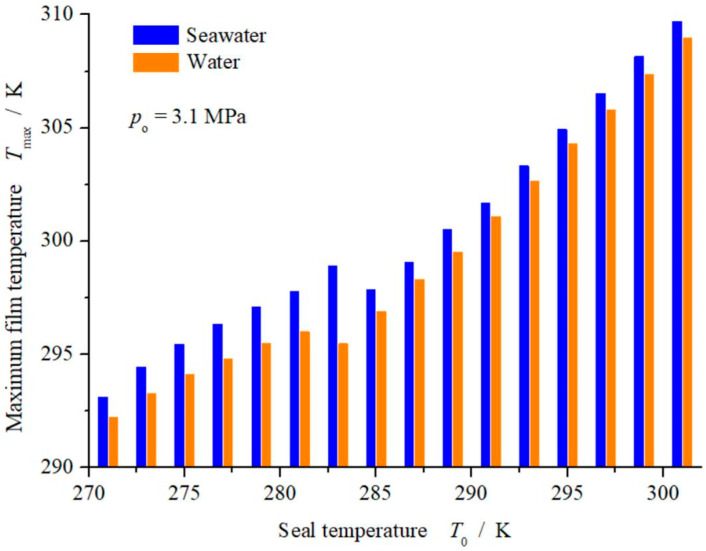
Variation in maximum film temperature with seal temperature (*w* = 10,000 rpm).

**Figure 15:**
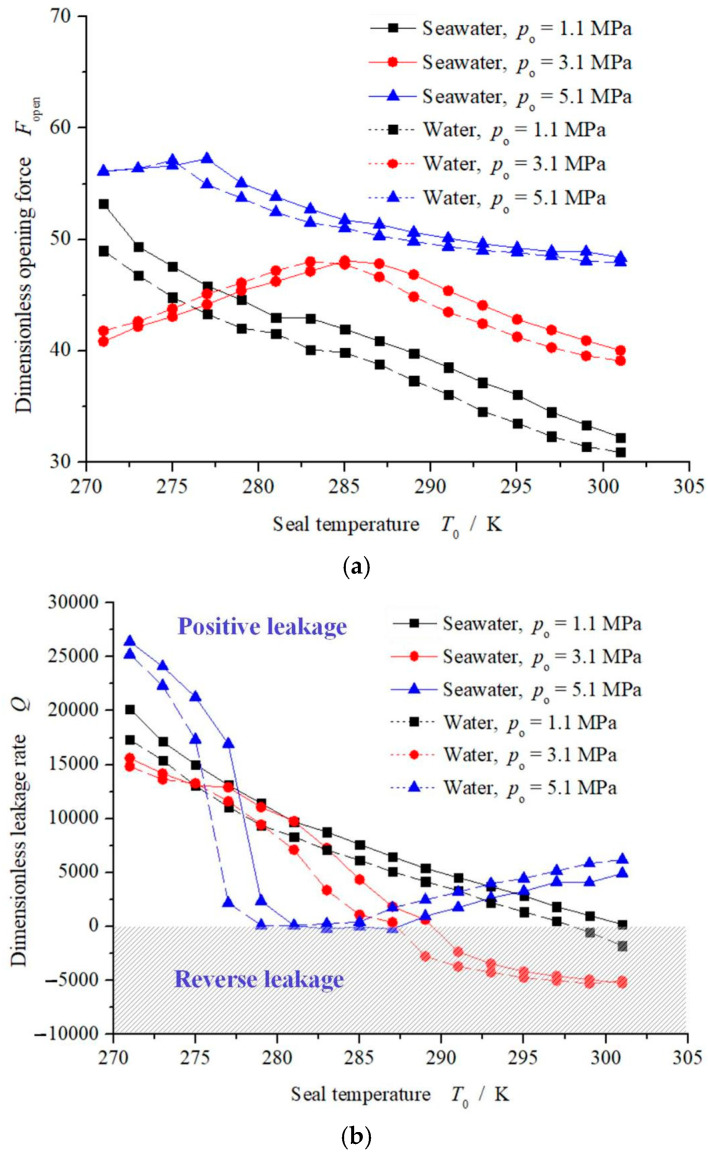
Variation in sealing performance with seal temperature. (**a**) Opening force and (**b**) leakage rate (*w* = 10,000 rpm).

Figure 14 illustrates the maximum film temperature at different seal temperatures. As expected, the film temperature increases with increasing seal temperature and is several degrees higher in seawater seals than in water. Figure 15 illustrates the impact of seal temperature on sealing performance. Overall, the seawater seal presents similar trends with water seals with increasing seal temperature. However, there are significant differences in the opening force and leakage rate values between seawater seals and water seals, at approximately 10%, which cannot be ignored when designing the reliability of the seal.

More importantly, not only does the seal temperature significantly affect the sealing performance, but also this influence is not always monotonic. For the seawater seal working at a seal pressure of *p*_o_ = 1.1 MPa, the opening force linearly decreases from 53 to 32, about a 40% reduction, when seal temperature increases from 273 K to 303 K. Theoretically, this will lead to contact wear of the sealing faces. However, for the higher-pressure case of *p*_o_ = 3.1 MPa, the opening force linearly increases from 42 at a seal temperature of 273 K to a peak value of 48 at a seal temperature of 285 K and then drops with a further increase in seal temperature. This 15% increase in opening force often leads to an increase in the sealing gap, accompanied by a sharp rise in the leakage rate. These are unacceptable in field applications.

One important reason lies in the fact that the change in temperature leads to the alteration of the properties of seawater fluid, which couples with the pumping effects of grooves and face deformations, ultimately presenting these complex changes. Further, with the increase in seal temperature, the leakage rate presents a transformation between positive and negative, which indicates that the seal is in a completely zero-leakage state within a certain range of ambient temperature.

### 5.2. Seal Pressure

**Figure 16:**
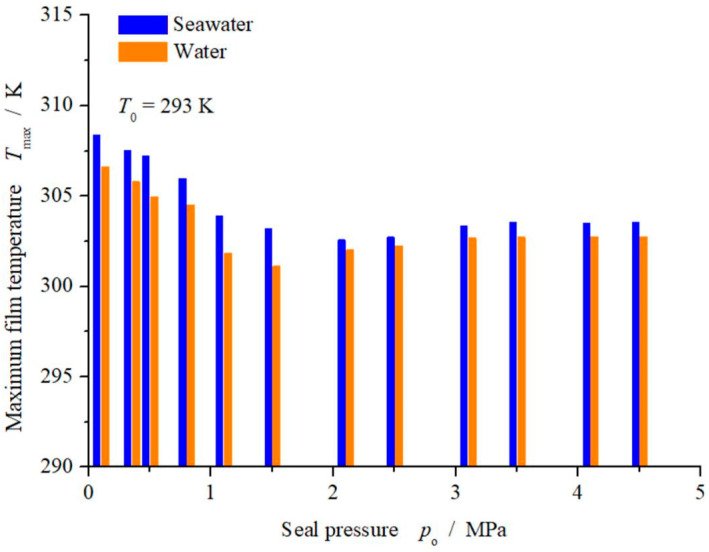
Variation in maximum film temperature with seal pressure (*w* = 10,000 rpm).

Seal pressure is another critical parameter that affects sealing performance. Figure 16 illustrates how the maximum film temperature varies with increasing seal pressure. Overall, as seal pressure increases from 0 to 4.5 MPa, there is a corresponding decrease in film temperature. However, because the temperature increase in the seal is not significant, the impact of seal pressure on the maximum film temperature is only a few degrees.

**Figure 17:**
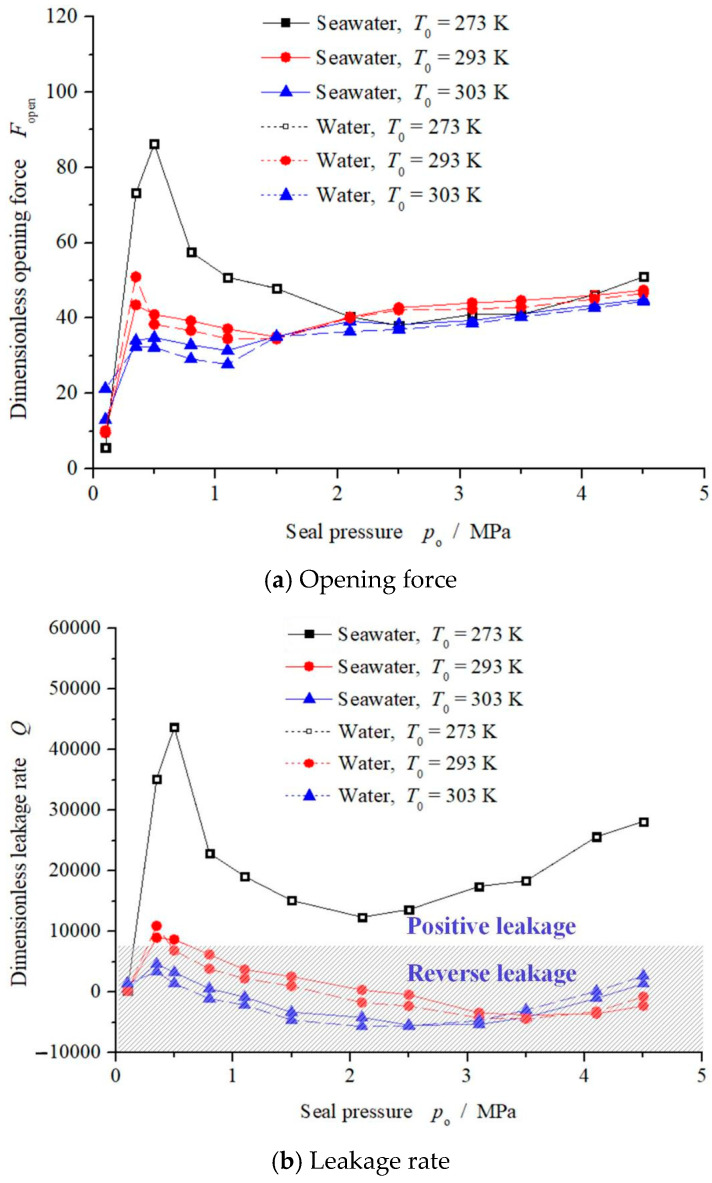
Variation in sealing performance with seal pressure (*w* = 10,000 rpm).

Generally, the opening force increases significantly with an increase in the seal pressure, without considering face deformation. Once face deformations are considered, the opening force may present a complex variation. Figure 17 illustrates the change in sealing performance with increasing seal pressure. An important point to note is that, when the seal pressure exceeds 2 MPa, the opening force appears to no longer change significantly with increasing seal pressure. The reason for this phenomenon may be that the face deformation induced by seal pressure counteracts the hydrodynamic effect induced by the outer spiral grooves. Correspondingly, there exists a seal pressure range of low leakage, as shown in Figure 17.

### 5.3. Speed

**Figure 18:**
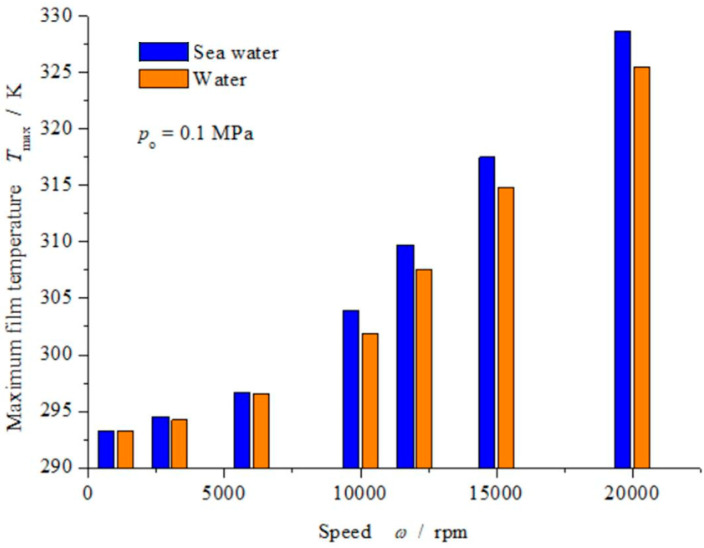
Variation in maximum film temperature with speed (*T*_o_ = 293 K).

The shear effect of speed not only leads to the hydrodynamic effect causing the opening force to increase but also results in temperature increase. As illustrated in Figure 18, the maximum film temperature rises with increasing speed. The maximum film temperature for the seawater seal increases from 293 K to 328 K, when the speed increases from 0 to 20,000 rpm.

As shown in Figure 18, the increase in speed can also lead to a significant increase in the opening force. However, this increase is nonlinear, especially for the working condition of *p*_o_ = 2.1 MPa, where a downward trend occurs after the speed exceeds 12,000 rpm.

**Figure 19:**
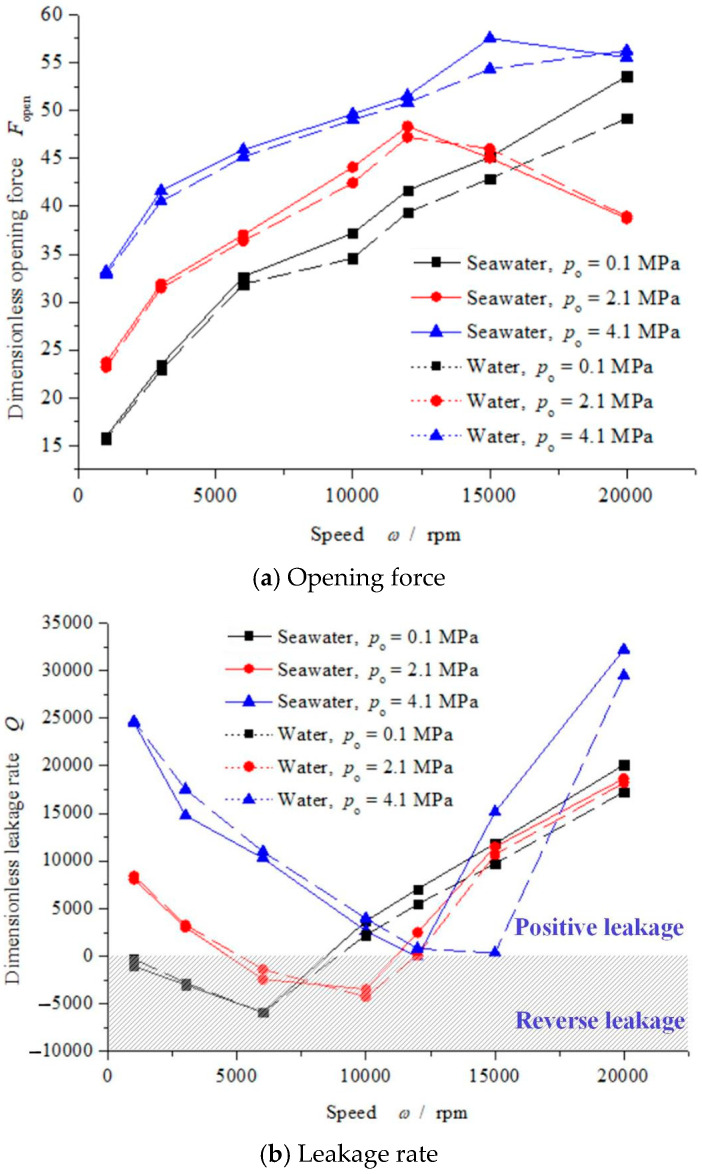
Variation in sealing performance with speed (*T*_o_ = 293 K).

It should also be noted that speed ranges exist for zero leakage, as shown in Figure 19. For the case of *p*_o_ = 0.1 MPa, the range is from 0 to 7500 rpm, whereas for the case of *p*_o_ = 2.1 MPa, the range is from 5000 rpm to 12,000 rpm. Based on the above analysis, the seal can achieve a zero-leakage design under multiple seal pressures, ambient temperatures and speeds.

Under high-speed working conditions, thermal deformation of the seal face caused by a temperature rise is an important factor leading to fluctuations in sealing performance. In practical engineering applications, enhancing the thermal conductivity of the sealing ring material and reducing the temperature gradient of the sealing ring can, to a certain extent, control the deformation of the sealing end face, thereby reducing the fluctuation of the sealing opening force and leakage rate.

### 5.4. Leakage Map

**Figure 20:**
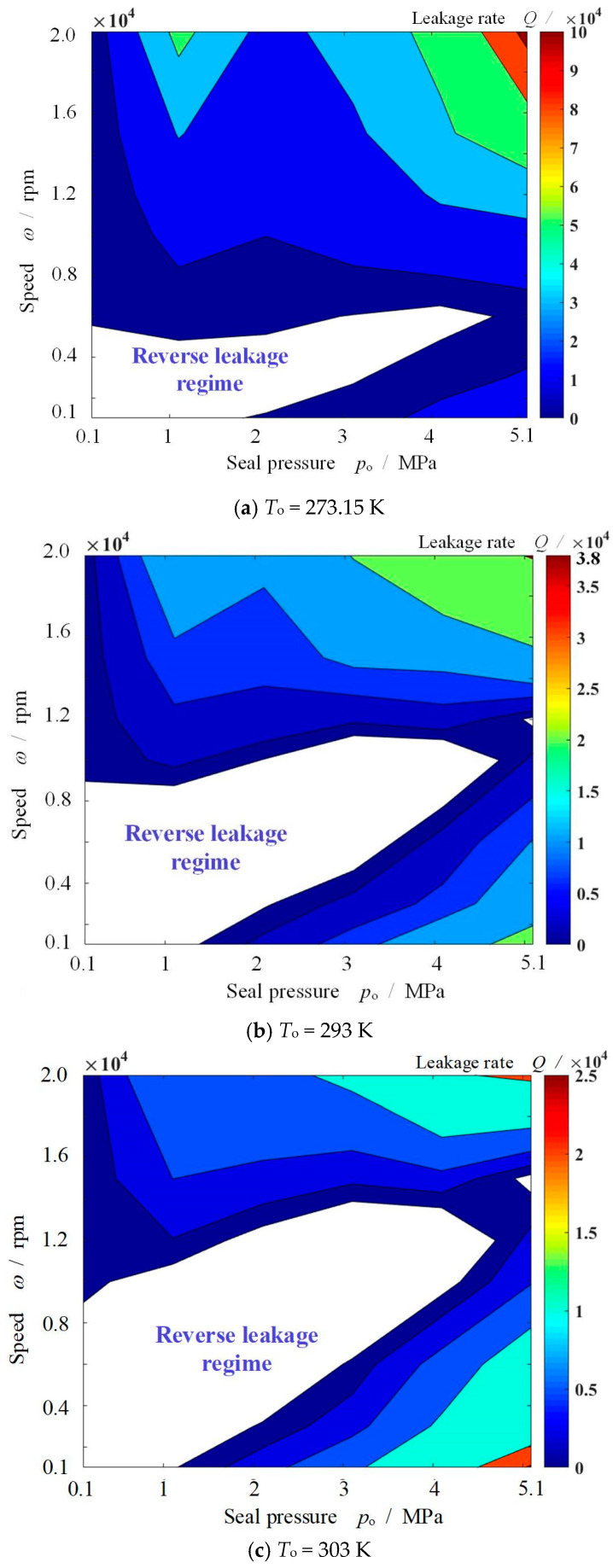
Zero-leakage map for multiple working conditions of pressure and speed.

To further clarify the leakage state, the contour of the leakage as a function of seal pressure and rotation speed is presented in Figure 20. In practical applications, the reverse leakage rate denotes a zero-leakage rate of the sealed fluid. As is clearly shown in the figure, there is a regime where the seal is in the state of zero leakage, when the speed is lower than 6000 rpm and the seal pressure is lower than 4.5 MPa at a temperature of 273 K. Obviously, the zero-leakage regime is located in the low-speed zone in the map. The primary reason for this is that the pumping effect induced by the inner grooves is less pronounced than that induced by the outer grooves.

Another important conclusion is that the reverse leakage regime in the map significantly expands with increasing ambient temperature. Meanwhile, the maximum speed in the reverse leakage regime increases from 6000 rpm to 13,000 rpm with the ambient temperature increasing from 273.15 K to 303 K. The maximum value of the leakage rate decreases from about 10 × 10^+4^ to 2.5 × 10^+4^. The reason for this is that, with the increase in temperature, the viscosity decreases, as seen in Figure 3, and a more obvious convergent deformation of the seal face is formed. This results in an increase in the shear rate of the inner groove, creating a more pronounced upstream pumping effect. Simultaneously, it leads to a reduction in the shear rate of the outer grooves, thereby weakening the pumping effect.

## 6. Conclusions

In this study, a TEHL model is proposed, taking into account the effects of salinity and cavitation. The TEHL analysis is carried out for the double spiral groove face seawater seal. Based on the numerical analysis results, the detailed conclusions can be summarized as follows:

(a) The effect of salinity on the sealing performance of seawater is evident, especially in the control of leakage rate. As salinity increases, the sealing performance undergoes complex changes, including nonlinear variations in opening force and leakage rate. When salinity increases from 0 to the standard 35 g/kg, the opening force varies by approximately 5%. Additionally, a transition between forward leakage and reverse leakage occurs, varying by approximately ±100% and resulting in leakage instability.
(b) The double spiral groove face seal combines the dynamic pressure effect of the outer groove with the upstream pumping effect of the inner groove and makes it possible to achieve zero leakage for seawater face seals, even at pressures exceeding 4 MPa, effectively enhancing the stability and reliability of the seal.
(c) Ambient temperature, rotational speed and sea pressure all significantly affect the leakage rate, but salinity does not. The TEHL analysis indicates that the double spiral groove demonstrates excellent adaptability under multi-point conditions and can help achieve a zero-leakage design under variable pressure, speed and temperature conditions. This provides theoretical support for deep-sea equipment and applications in other extreme environments.

## Figures and Tables

**Table 1:** Characteristics of the sealing materials [8].

Properties	Carbon	Silicon Carbide
Density/kg·m^−3^	1800	3100
Young’s modulus/GPa	25	400
Poisson’s ratio	0.20	0.17
Specific heat capacity/J·kg^−1^·K^−1^	710	400
Thermal conductivity/W·m^−1^·K^−1^	15	150
Coefficient of thermal expansion/10^−6^ K^−1^	4	4.3

## Data Availability

The original contributions presented in this study are included in the article. Further inquiries can be directed to the corresponding author.
